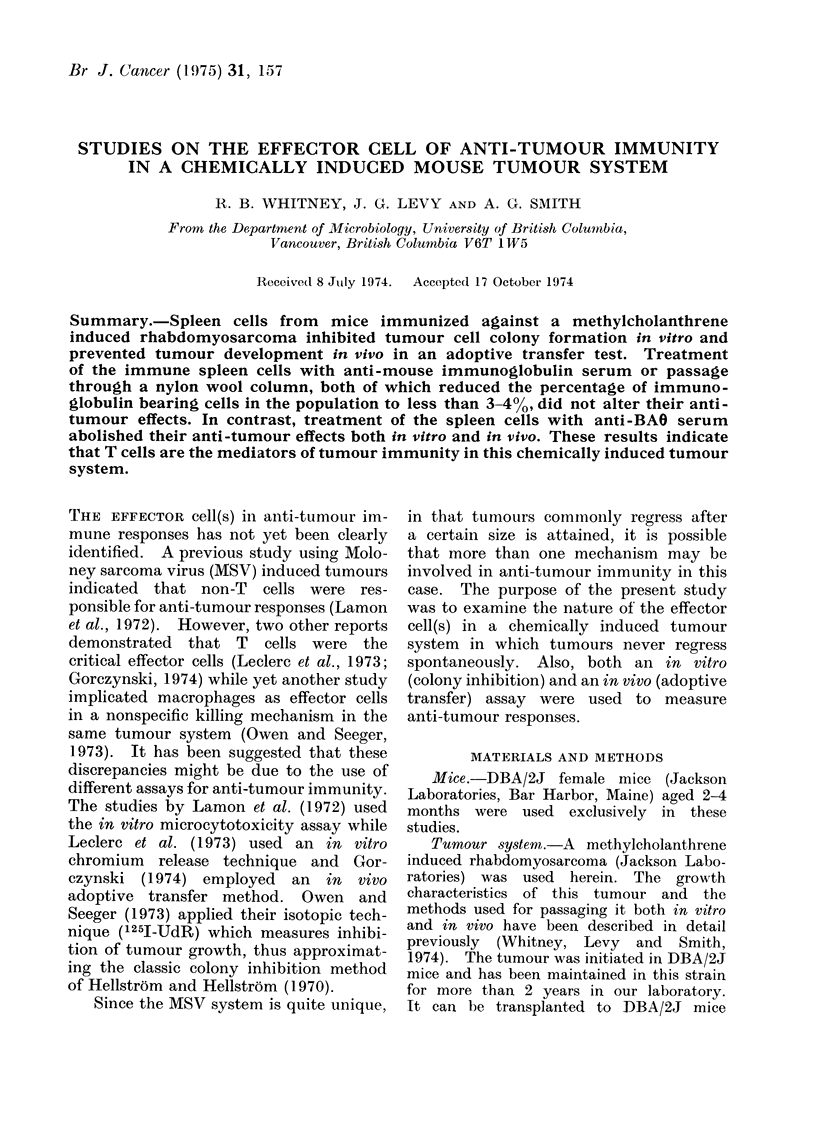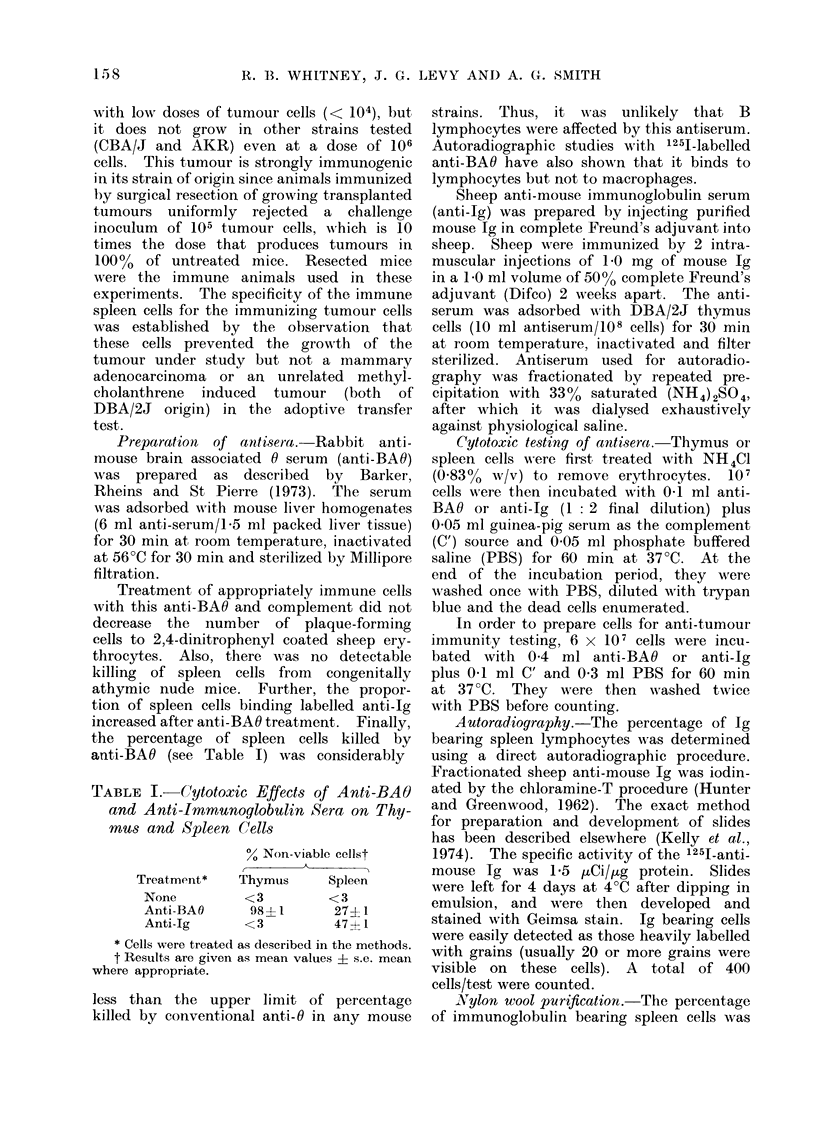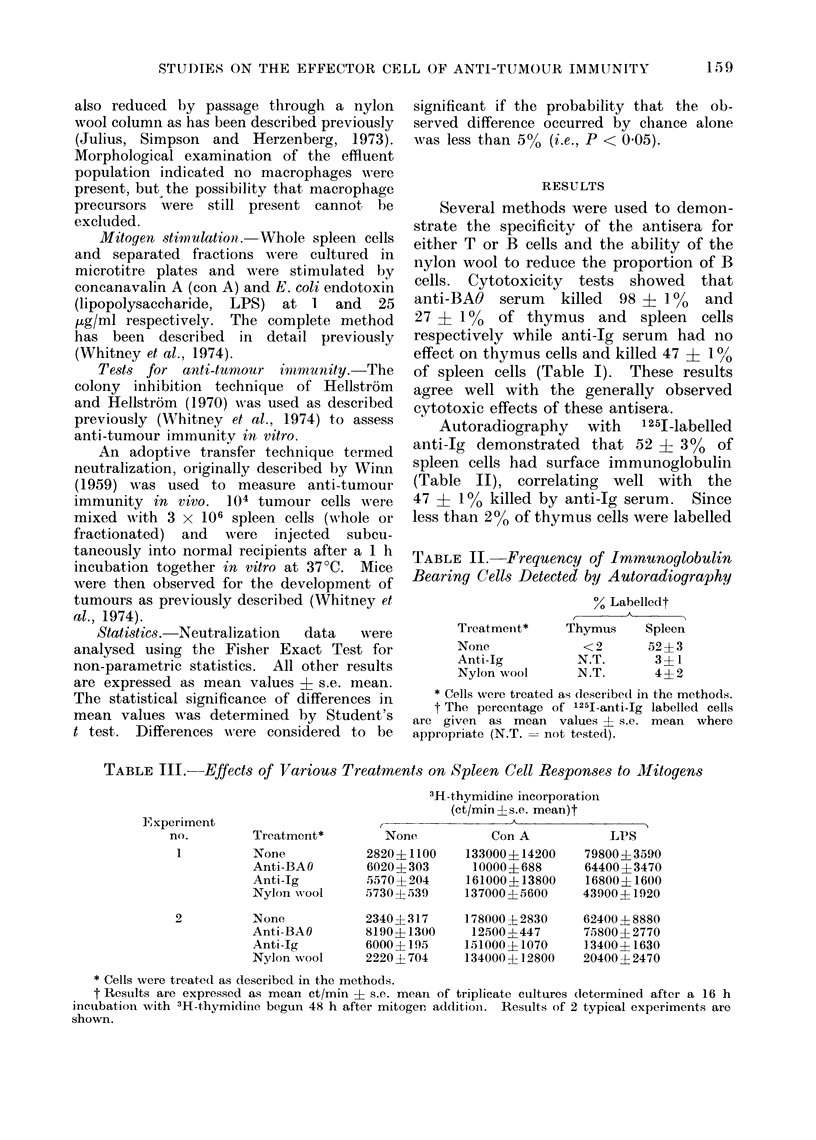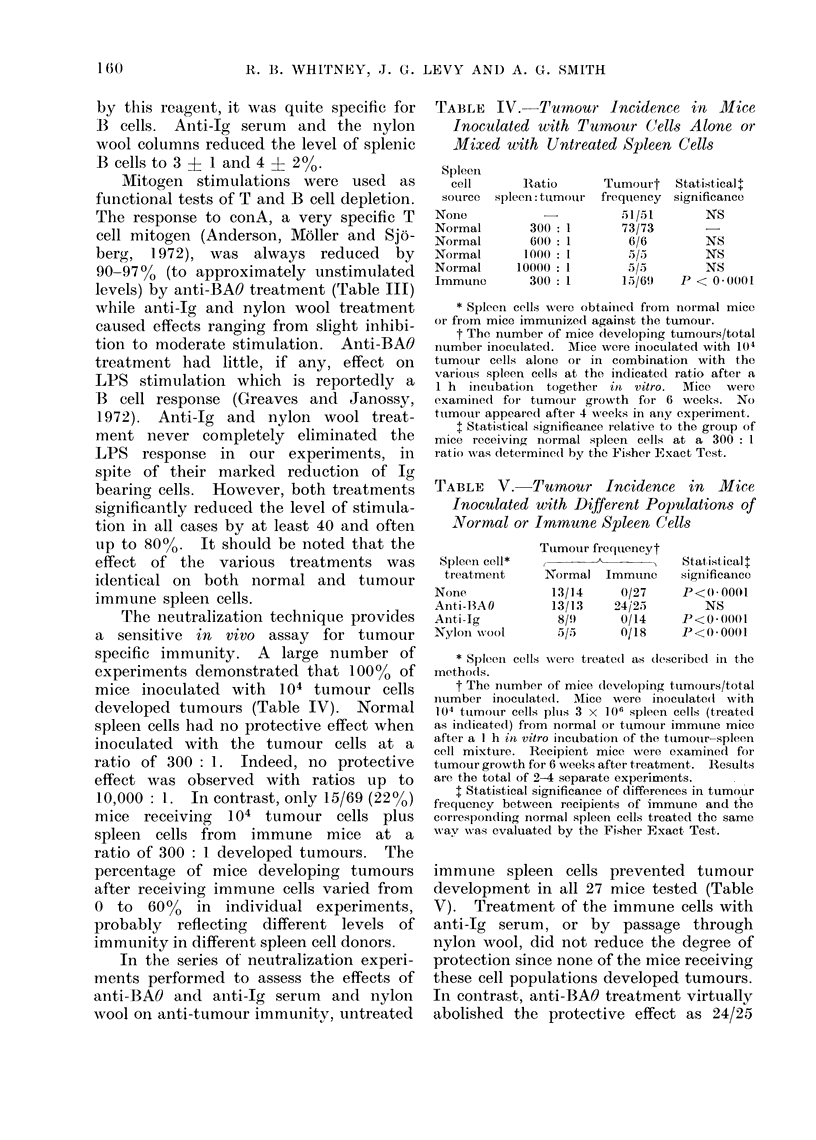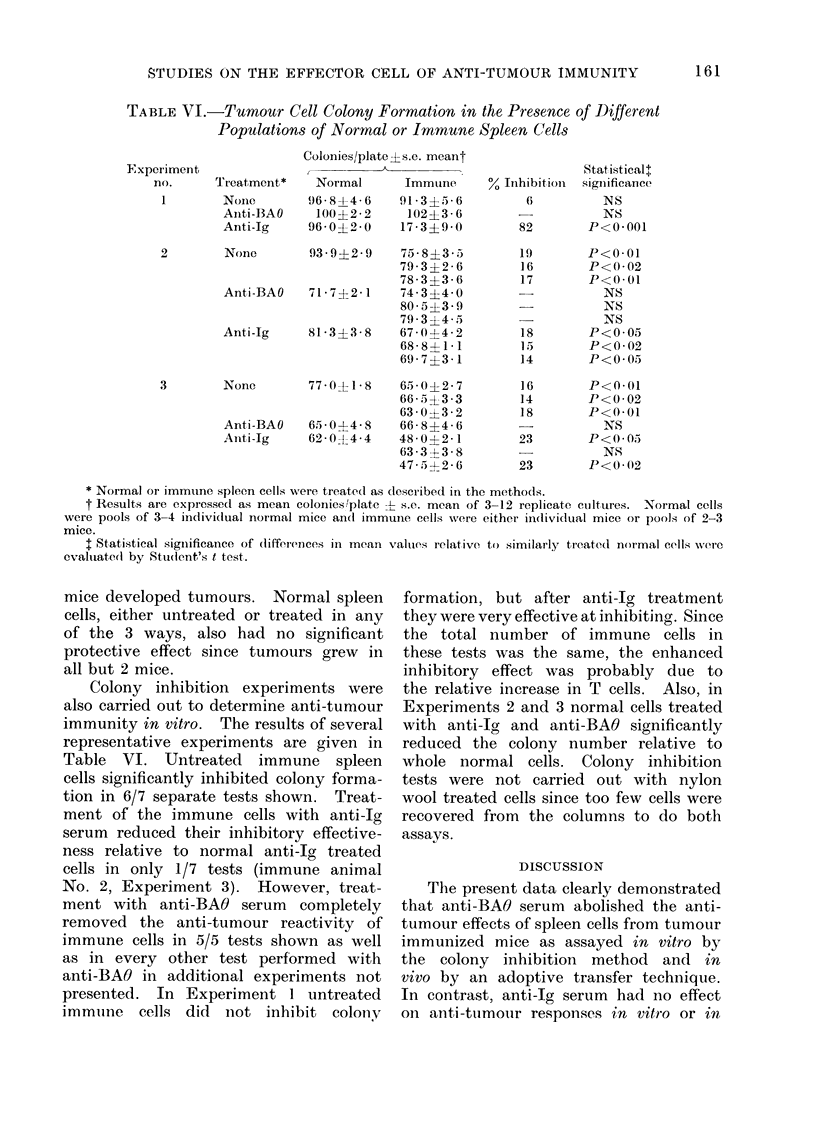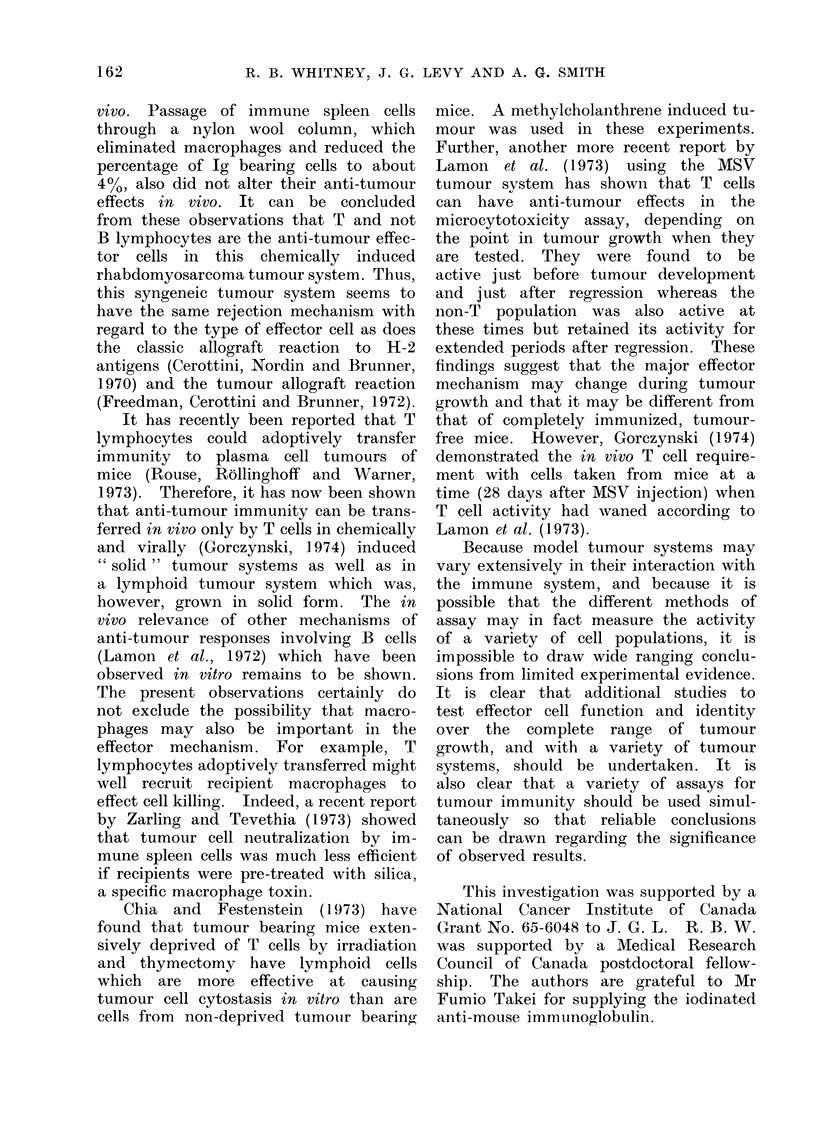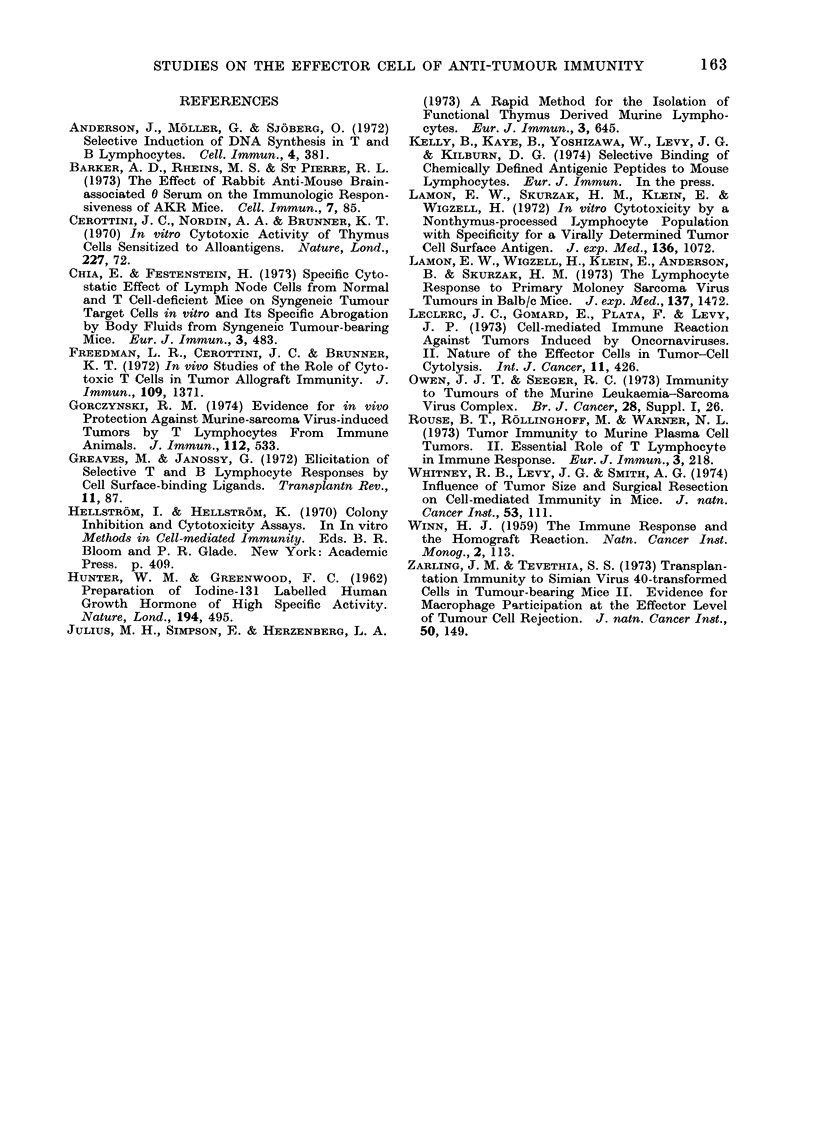# Studies on the effector cell of anti-tumour immunity in a chemically induced mouse tumour system.

**DOI:** 10.1038/bjc.1975.20

**Published:** 1975-02

**Authors:** R. B. Whitney, J. G. Levy, A. G. Smith

## Abstract

Spleen cells from mice immunized against a methylcholanthrene induced rhabdomyosarcoma inhibited tumour cell colony formation in vitro and prevented tumour development in vivo in an adoptive transfer test. Treatment of the immune spleen cells with anti-mouse immunoglobulin serum or passage through a nylon wool column, both of which reduced the percentage of immunoglobulin bearing cells in the population to less than 3-4%, did not alter their anti-tumour effects. In contrast, treatment of the spleen cells with anti-BAomicron serum abolished their anti-tumour effects both in vitro and in vivo. These results indicate that T cells are the mediators of tumour immunity in this chemically induced tumour system.


*Br J. Cancer* (1975) **31**, 157

### STUDIES ON THE EFFECTOR CELL OF ANTI-TUMOUR IMMUNITY IN A CHEMICALLY INDUCED MOUSE TUMOUR SYSTEM

R. B. WHITNEY, J. G. LEVY AND A. G. SMITH

*From the Department of Microbiology, University of British Columbia, Vancouver, British Columbia V6T 1W5*

Received 8 July 1974. Accepted 17 October 1974

**Summary.—Spleen cells from mice immunized against a methylcholanthrene induced rhabdomyosarcoma inhibited tumour cell colony formation *in vitro* and prevented tumour development *in vivo* in an adoptive transfer test. Treatment of the immune spleen cells with anti-mouse immunoglobulin serum or passage through a nylon wool column, both of which reduced the percentage of immunoglobulin bearing cells in the population to less than 3–4%, did not alter their anti-tumour effects. In contrast, treatment of the spleen cells with anti-BAθ serum abolished their anti-tumour effects both *in vitro* and *in vivo*. These results indicate that T cells are the mediators of tumour immunity in this chemically induced tumour system.**

THE EFFECTOR cell(s) in anti-tumour immune responses has not yet been clearly identified. A previous study using Moloney sarcoma virus (MSV) induced tumours indicated that non-T cells were responsible for anti-tumour responses (Lamon *et al.*, 1972). However, two other reports demonstrated that T cells were the critical effector cells (Leclerc *et al.*, 1973; Gorczynski, 1974) while yet another study implicated macrophages as effector cells in a nonspecific killing mechanism in the same tumour system (Owen and Seeger, 1973). It has been suggested that these discrepancies might be due to the use of different assays for anti-tumour immunity. The studies by Lamon *et al.* (1972) used the *in vitro* microcytotoxicity assay while Leclerc *et al.* (1973) used an *in vitro* chromium release technique and Gorczynski (1974) employed an *in vivo* adoptive transfer method. Owen and Seeger (1973) applied their isotopic technique ($^{125}$I-UdR) which measures inhibition of tumour growth, thus approximating the classic colony inhibition method of Hellström and Hellström (1970).

Since the MSV system is quite unique, in that tumours commonly regress after a certain size is attained, it is possible that more than one mechanism may be involved in anti-tumour immunity in this case. The purpose of the present study was to examine the nature of the effector cell(s) in a chemically induced tumour system in which tumours never regress spontaneously. Also, both an *in vitro* (colony inhibition) and an *in vivo* (adoptive transfer) assay were used to measure anti-tumour responses.

### MATERIALS AND METHODS

*Mice.*—DBA/2J female mice (Jackson Laboratories, Bar Harbor, Maine) aged 2–4 months were used exclusively in these studies.

*Tumour system.*—A methylcholanthrene induced rhabdomyosarcoma (Jackson Laboratories) was used herein. The growth characteristics of this tumour and the methods used for passaging it both *in vitro* and *in vivo* have been described in detail previously (Whitney, Levy and Smith, 1974). The tumour was initiated in DBA/2J mice and has been maintained in this strain for more than 2 years in our laboratory. It can be transplanted to DBA/2J mice with low doses of tumour cells ($< 10^4$), but it does not grow in other strains tested (CBA/J and AKR) even at a dose of $10^6$ cells. This tumour is strongly immunogenic in its strain of origin since animals immunized by surgical resection of growing transplanted tumours uniformly rejected a challenge inoculum of $10^5$ tumour cells, which is 10 times the dose that produces tumours in 100% of untreated mice. Resected mice were the immune animals used in these experiments. The specificity of the immune spleen cells for the immunizing tumour cells was established by the observation that these cells prevented the growth of the tumour under study but not a mammary adenocarcinoma or an unrelated methylcholanthrene induced tumour (both of DBA/2J origin) in the adoptive transfer test.

*Preparation of antisera.*—Rabbit anti-mouse brain associated $\theta$ serum (anti-BA$\theta$) was prepared as described by Barker, Rheins and St Pierre (1973). The serum was adsorbed with mouse liver homogenates (6 ml anti-serum/1·5 ml packed liver tissue) for 30 min at room temperature, inactivated at 56°C for 30 min and sterilized by Millipore filtration.

Treatment of appropriately immune cells with this anti-BA$\theta$ and complement did not decrease the number of plaque-forming cells to 2,4-dinitrophenyl coated sheep erythrocytes. Also, there was no detectable killing of spleen cells from congenitally athymic nude mice. Further, the proportion of spleen cells binding labelled anti-Ig increased after anti-BA$\theta$ treatment. Finally, the percentage of spleen cells killed by anti-BA$\theta$ (see Table I) was considerably less than the upper limit of percentage killed by conventional anti-$\theta$ in any mouse strains. Thus, it was unlikely that B lymphocytes were affected by this antiserum. Autoradiographic studies with $^{125}$I-labelled anti-BA$\theta$ have also shown that it binds to lymphocytes but not to macrophages.

TABLE I.—*Cytotoxic Effects of Anti-BA$\theta$ and Anti-Immunoglobulin Sera on Thymus and Spleen Cells*

| | % Non-viable cells† | |
|---|---|---|
| Treatment* | Thymus | Spleen |
| None | <3 | <3 |
| Anti-BA$\theta$ | 98±1 | 27±1 |
| Anti-Ig | <3 | 47±1 |

* Cells were treated as described in the methods.
† Results are given as mean values ± s.e. mean where appropriate.

Sheep anti-mouse immunoglobulin serum (anti-Ig) was prepared by injecting purified mouse Ig in complete Freund's adjuvant into sheep. Sheep were immunized by 2 intramuscular injections of 1·0 mg of mouse Ig in a 1·0 ml volume of 50% complete Freund's adjuvant (Difco) 2 weeks apart. The antiserum was adsorbed with DBA/2J thymus cells (10 ml antiserum/$10^8$ cells) for 30 min at room temperature, inactivated and filter sterilized. Antiserum used for autoradiography was fractionated by repeated precipitation with 33% saturated $(NH_4)_2SO_4$, after which it was dialysed exhaustively against physiological saline.

*Cytotoxic testing of antisera.*—Thymus or spleen cells were first treated with $NH_4Cl$ (0·83% w/v) to remove erythrocytes. $10^7$ cells were then incubated with 0·1 ml anti-BA$\theta$ or anti-Ig (1 : 2 final dilution) plus 0·05 ml guinea-pig serum as the complement (C′) source and 0·05 ml phosphate buffered saline (PBS) for 60 min at 37°C. At the end of the incubation period, they were washed once with PBS, diluted with trypan blue and the dead cells enumerated.

In order to prepare cells for anti-tumour immunity testing, $6 \times 10^7$ cells were incubated with 0·4 ml anti-BA$\theta$ or anti-Ig plus 0·1 ml C′ and 0·3 ml PBS for 60 min at 37°C. They were then washed twice with PBS before counting.

*Autoradiography.*—The percentage of Ig bearing spleen lymphocytes was determined using a direct autoradiographic procedure. Fractionated sheep anti-mouse Ig was iodinated by the chloramine-T procedure (Hunter and Greenwood, 1962). The exact method for preparation and development of slides has been described elsewhere (Kelly *et al.*, 1974). The specific activity of the $^{125}$I-anti-mouse Ig was 1·5 $\mu$Ci/$\mu$g protein. Slides were left for 4 days at 4°C after dipping in emulsion, and were then developed and stained with Geimsa stain. Ig bearing cells were easily detected as those heavily labelled with grains (usually 20 or more grains were visible on these cells). A total of 400 cells/test were counted.

*Nylon wool purification.*—The percentage of immunoglobulin bearing spleen cells was also reduced by passage through a nylon wool column as has been described previously (Julius, Simpson and Herzenberg, 1973). Morphological examination of the effluent population indicated no macrophages were present, but the possibility that macrophage precursors were still present cannot be excluded.

*Mitogen stimulation.*—Whole spleen cells and separated fractions were cultured in microtitre plates and were stimulated by concanavalin A (con A) and *E. coli* endotoxin (lipopolysaccharide, LPS) at 1 and 25 μg/ml respectively. The complete method has been described in detail previously (Whitney *et al.*, 1974).

*Tests for anti-tumour immunity.*—The colony inhibition technique of Hellström and Hellström (1970) was used as described previously (Whitney *et al.*, 1974) to assess anti-tumour immunity *in vitro.*

An adoptive transfer technique termed neutralization, originally described by Winn (1959) was used to measure anti-tumour immunity *in vivo.* $10^4$ tumour cells were mixed with $3 \times 10^6$ spleen cells (whole or fractionated) and were injected subcutaneously into normal recipients after a 1 h incubation together *in vitro* at 37°C. Mice were then observed for the development of tumours as previously described (Whitney *et al.*, 1974).

*Statistics.*—Neutralization data were analysed using the Fisher Exact Test for non-parametric statistics. All other results are expressed as mean values ± s.e. mean. The statistical significance of differences in mean values was determined by Student's *t* test. Differences were considered to be significant if the probability that the observed difference occurred by chance alone was less than 5% (*i.e.*, $P < 0{\cdot}05$).

### RESULTS

Several methods were used to demonstrate the specificity of the antisera for either T or B cells and the ability of the nylon wool to reduce the proportion of B cells. Cytotoxicity tests showed that anti-BA$\theta$ serum killed 98 ± 1% and 27 ± 1% of thymus and spleen cells respectively while anti-Ig serum had no effect on thymus cells and killed 47 ± 1% of spleen cells (Table I). These results agree well with the generally observed cytotoxic effects of these antisera.

Autoradiography with $^{125}$I-labelled anti-Ig demonstrated that 52 ± 3% of spleen cells had surface immunoglobulin (Table II), correlating well with the 47 ± 1% killed by anti-Ig serum. Since less than 2% of thymus cells were labelled

TABLE II.—*Frequency of Immunoglobulin Bearing Cells Detected by Autoradiography*

| | % Labelled† | |
|---|---|---|
| Treatment* | Thymus | Spleen |
| None | <2 | 52±3 |
| Anti-Ig | N.T. | 3±1 |
| Nylon wool | N.T. | 4±2 |

* Cells were treated as described in the methods.
† The percentage of $^{125}$I-anti-Ig labelled cells are given as mean values ± s.e. mean where appropriate (N.T. = not tested).

TABLE III.—*Effects of Various Treatments on Spleen Cell Responses to Mitogens*

| | | $^3$H-thymidine incorporation (ct/min ± s.e. mean)† | | |
|---|---|---|---|---|
| Experiment no. | Treatment* | None | Con A | LPS |
| 1 | None | 2820±1100 | 133000±14200 | 79800±3590 |
| | Anti-BA$\theta$ | 6020±303 | 10000±688 | 64400±3470 |
| | Anti-Ig | 5570±204 | 161000±13800 | 16800±1600 |
| | Nylon wool | 5730±539 | 137000±5600 | 43900±1920 |
| 2 | None | 2340±317 | 178000±2830 | 62400±8880 |
| | Anti-BA$\theta$ | 8190±1300 | 12500±447 | 75800±2770 |
| | Anti-Ig | 6000±195 | 151000±1070 | 13400±1630 |
| | Nylon wool | 2220±704 | 134000±12800 | 20400±2470 |

* Cells were treated as described in the methods.
† Results are expressed as mean ct/min ± s.e. mean of triplicate cultures determined after a 16 h incubation with $^3$H-thymidine begun 48 h after mitogen addition. Results of 2 typical experiments are shown.

by this reagent, it was quite specific for B cells. Anti-Ig serum and the nylon wool columns reduced the level of splenic B cells to 3 ± 1 and 4 ± 2%.

Mitogen stimulations were used as functional tests of T and B cell depletion. The response to conA, a very specific T cell mitogen (Anderson, Möller and Sjöberg, 1972), was always reduced by 90–97% (to approximately unstimulated levels) by anti-BA$\theta$ treatment (Table III) while anti-Ig and nylon wool treatment caused effects ranging from slight inhibition to moderate stimulation. Anti-BA$\theta$ treatment had little, if any, effect on LPS stimulation which is reportedly a B cell response (Greaves and Janossy, 1972). Anti-Ig and nylon wool treatment never completely eliminated the LPS response in our experiments, in spite of their marked reduction of Ig bearing cells. However, both treatments significantly reduced the level of stimulation in all cases by at least 40 and often up to 80%. It should be noted that the effect of the various treatments was identical on both normal and tumour immune spleen cells.

The neutralization technique provides a sensitive *in vivo* assay for tumour specific immunity. A large number of experiments demonstrated that 100% of mice inoculated with $10^4$ tumour cells developed tumours (Table IV). Normal spleen cells had no protective effect when inoculated with the tumour cells at a ratio of 300 : 1. Indeed, no protective effect was observed with ratios up to 10,000 : 1. In contrast, only 15/69 (22%) mice receiving $10^4$ tumour cells plus spleen cells from immune mice at a ratio of 300 : 1 developed tumours. The percentage of mice developing tumours after receiving immune cells varied from 0 to 60% in individual experiments, probably reflecting different levels of immunity in different spleen cell donors.

In the series of neutralization experiments performed to assess the effects of anti-BA$\theta$ and anti-Ig serum and nylon wool on anti-tumour immunity, untreated immune spleen cells prevented tumour development in all 27 mice tested (Table V). Treatment of the immune cells with anti-Ig serum, or by passage through nylon wool, did not reduce the degree of protection since none of the mice receiving these cell populations developed tumours. In contrast, anti-BA$\theta$ treatment virtually abolished the protective effect as 24/25

TABLE IV.—*Tumour Incidence in Mice Inoculated with Tumour Cells Alone or Mixed with Untreated Spleen Cells*

| Spleen cell source | Ratio spleen : tumour | Tumour† frequency | Statistical‡ significance |
|---|---|---|---|
| None | — | 51/51 | NS |
| Normal | 300 : 1 | 73/73 | — |
| Normal | 600 : 1 | 6/6 | NS |
| Normal | 1000 : 1 | 5/5 | NS |
| Normal | 10000 : 1 | 5/5 | NS |
| Immune | 300 : 1 | 15/69 | $P < 0.0001$ |

* Spleen cells were obtained from normal mice or from mice immunized against the tumour.

† The number of mice developing tumours/total number inoculated. Mice were inoculated with $10^4$ tumour cells alone or in combination with the various spleen cells at the indicated ratio after a 1 h incubation together *in vitro*. Mice were examined for tumour growth for 6 weeks. No tumour appeared after 4 weeks in any experiment.

‡ Statistical significance relative to the group of mice receiving normal spleen cells at a 300 : 1 ratio was determined by the Fisher Exact Test.

TABLE V.—*Tumour Incidence in Mice Inoculated with Different Populations of Normal or Immune Spleen Cells*

| Spleen cell* treatment | Tumour frequency† | | Statistical‡ significance |
|---|---|---|---|
| | Normal | Immune | |
| None | 13/14 | 0/27 | $P<0.0001$ |
| Anti-BA$\theta$ | 13/13 | 24/25 | NS |
| Anti-Ig | 8/9 | 0/14 | $P<0.0001$ |
| Nylon wool | 5/5 | 0/18 | $P<0.0001$ |

* Spleen cells were treated as described in the methods.

† The number of mice developing tumours/total number inoculated. Mice were inoculated with $10^4$ tumour cells plus $3 \times 10^6$ spleen cells (treated as indicated) from normal or tumour immune mice after a 1 h *in vitro* incubation of the tumour–spleen cell mixture. Recipient mice were examined for tumour growth for 6 weeks after treatment. Results are the total of 2–4 separate experiments.

‡ Statistical significance of differences in tumour frequency between recipients of immune and the corresponding normal spleen cells treated the same way was evaluated by the Fisher Exact Test.

TABLE VI.—*Tumour Cell Colony Formation in the Presence of Different Populations of Normal or Immune Spleen Cells*

| Experiment no. | Treatment* | Colonies/plate ± s.e. mean† Normal | Immune | % Inhibition | Statistical‡ significance |
|---|---|---|---|---|---|
| 1 | None | 96·8±4·6 | 91·3±5·6 | 6 | NS |
| | Anti-BA$\theta$ | 100±2·2 | 102±3·6 | — | NS |
| | Anti-Ig | 96·0±2·0 | 17·3±9·0 | 82 | $P<0·001$ |
| 2 | None | 93·9±2·9 | 75·8±3·5 | 19 | $P<0·01$ |
| | | | 79·3±2·6 | 16 | $P<0·02$ |
| | | | 78·3±3·6 | 17 | $P<0·01$ |
| | Anti-BA$\theta$ | 71·7±2·1 | 74·3±4·0 | — | NS |
| | | | 80·5±3·9 | — | NS |
| | | | 79·3±4·5 | — | NS |
| | Anti-Ig | 81·3±3·8 | 67·0±4·2 | 18 | $P<0·05$ |
| | | | 68·8±1·1 | 15 | $P<0·02$ |
| | | | 69·7±3·1 | 14 | $P<0·05$ |
| 3 | None | 77·0±1·8 | 65·0±2·7 | 16 | $P<0·01$ |
| | | | 66·5±3·3 | 14 | $P<0·02$ |
| | | | 63·0±3·2 | 18 | $P<0·01$ |
| | Anti-BA$\theta$ | 65·0±4·8 | 66·8±4·6 | — | NS |
| | Anti-Ig | 62·0±4·4 | 48·0±2·1 | 23 | $P<0·05$ |
| | | | 63·3±3·8 | — | NS |
| | | | 47·5±2·6 | 23 | $P<0·02$ |

* Normal or immune spleen cells were treated as described in the methods.

† Results are expressed as mean colonies/plate ± s.e. mean of 3–12 replicate cultures. Normal cells were pools of 3–4 individual normal mice and immune cells were either individual mice or pools of 2–3 mice.

‡ Statistical significance of differences in mean values relative to similarly treated normal cells were evaluated by Student's $t$ test.

mice developed tumours. Normal spleen cells, either untreated or treated in any of the 3 ways, also had no significant protective effect since tumours grew in all but 2 mice.

Colony inhibition experiments were also carried out to determine anti-tumour immunity *in vitro*. The results of several representative experiments are given in Table VI. Untreated immune spleen cells significantly inhibited colony formation in 6/7 separate tests shown. Treatment of the immune cells with anti-Ig serum reduced their inhibitory effectiveness relative to normal anti-Ig treated cells in only 1/7 tests (immune animal No. 2, Experiment 3). However, treatment with anti-BA$\theta$ serum completely removed the anti-tumour reactivity of immune cells in 5/5 tests shown as well as in every other test performed with anti-BA$\theta$ in additional experiments not presented. In Experiment 1 untreated immune cells did not inhibit colony formation, but after anti-Ig treatment they were very effective at inhibiting. Since the total number of immune cells in these tests was the same, the enhanced inhibitory effect was probably due to the relative increase in T cells. Also, in Experiments 2 and 3 normal cells treated with anti-Ig and anti-BA$\theta$ significantly reduced the colony number relative to whole normal cells. Colony inhibition tests were not carried out with nylon wool treated cells since too few cells were recovered from the columns to do both assays.

### DISCUSSION

The present data clearly demonstrated that anti-BA$\theta$ serum abolished the anti-tumour effects of spleen cells from tumour immunized mice as assayed *in vitro* by the colony inhibition method and *in vivo* by an adoptive transfer technique. In contrast, anti-Ig serum had no effect on anti-tumour responses *in vitro* or *in*

*vivo*. Passage of immune spleen cells through a nylon wool column, which eliminated macrophages and reduced the percentage of Ig bearing cells to about 4%, also did not alter their anti-tumour effects *in vivo*. It can be concluded from these observations that T and not B lymphocytes are the anti-tumour effector cells in this chemically induced rhabdomyosarcoma tumour system. Thus, this syngeneic tumour system seems to have the same rejection mechanism with regard to the type of effector cell as does the classic allograft reaction to H-2 antigens (Cerottini, Nordin and Brunner, 1970) and the tumour allograft reaction (Freedman, Cerottini and Brunner, 1972).

It has recently been reported that T lymphocytes could adoptively transfer immunity to plasma cell tumours of mice (Rouse, Röllinghoff and Warner, 1973). Therefore, it has now been shown that anti-tumour immunity can be transferred *in vivo* only by T cells in chemically and virally (Gorczynski, 1974) induced " solid " tumour systems as well as in a lymphoid tumour system which was, however, grown in solid form. The *in vivo* relevance of other mechanisms of anti-tumour responses involving B cells (Lamon *et al.*, 1972) which have been observed *in vitro* remains to be shown. The present observations certainly do not exclude the possibility that macrophages may also be important in the effector mechanism. For example, T lymphocytes adoptively transferred might well recruit recipient macrophages to effect cell killing. Indeed, a recent report by Zarling and Tevethia (1973) showed that tumour cell neutralization by immune spleen cells was much less efficient if recipients were pre-treated with silica, a specific macrophage toxin.

Chia and Festenstein (1973) have found that tumour bearing mice extensively deprived of T cells by irradiation and thymectomy have lymphoid cells which are more effective at causing tumour cell cytostasis *in vitro* than are cells from non-deprived tumour bearing mice. A methylcholanthrene induced tumour was used in these experiments. Further, another more recent report by Lamon *et al.* (1973) using the MSV tumour system has shown that T cells can have anti-tumour effects in the microcytotoxicity assay, depending on the point in tumour growth when they are tested. They were found to be active just before tumour development and just after regression whereas the non-T population was also active at these times but retained its activity for extended periods after regression. These findings suggest that the major effector mechanism may change during tumour growth and that it may be different from that of completely immunized, tumour-free mice. However, Gorczynski (1974) demonstrated the *in vivo* T cell requirement with cells taken from mice at a time (28 days after MSV injection) when T cell activity had waned according to Lamon *et al.* (1973).

Because model tumour systems may vary extensively in their interaction with the immune system, and because it is possible that the different methods of assay may in fact measure the activity of a variety of cell populations, it is impossible to draw wide ranging conclusions from limited experimental evidence. It is clear that additional studies to test effector cell function and identity over the complete range of tumour growth, and with a variety of tumour systems, should be undertaken. It is also clear that a variety of assays for tumour immunity should be used simultaneously so that reliable conclusions can be drawn regarding the significance of observed results.

This investigation was supported by a National Cancer Institute of Canada Grant No. 65-6048 to J. G. L. R. B. W. was supported by a Medical Research Council of Canada postdoctoral fellowship. The authors are grateful to Mr Fumio Takei for supplying the iodinated anti-mouse immunoglobulin.

### REFERENCES

ANDERSON, J., MÖLLER, G. & SJÖBERG, O. (1972) Selective Induction of DNA Synthesis in T and B Lymphocytes. *Cell. Immun.*, **4**, 381.

BARKER, A. D., RHEINS, M. S. & ST PIERRE, R. L. (1973) The Effect of Rabbit Anti-Mouse Brain-associated $\theta$ Serum on the Immunologic Responsiveness of AKR Mice. *Cell. Immun.*, **7**, 85.

CEROTTINI, J. C., NORDIN, A. A. & BRUNNER, K. T. (1970) *In vitro* Cytotoxic Activity of Thymus Cells Sensitized to Alloantigens. *Nature, Lond.*, **227**, 72.

CHIA, E. & FESTENSTEIN, H. (1973) Specific Cytostatic Effect of Lymph Node Cells from Normal and T Cell-deficient Mice on Syngeneic Tumour Target Cells *in vitro* and Its Specific Abrogation by Body Fluids from Syngeneic Tumour-bearing Mice. *Eur. J. Immun.*, **3**, 483.

FREEDMAN, L. R., CEROTTINI, J. C. & BRUNNER, K. T. (1972) *In vivo* Studies of the Role of Cytotoxic T Cells in Tumor Allograft Immunity. *J. Immun.*, **109**, 1371.

GORCZYNSKI, R. M. (1974) Evidence for *in vivo* Protection Against Murine-sarcoma Virus-induced Tumors by T Lymphocytes From Immune Animals. *J. Immun.*, **112**, 533.

GREAVES, M. & JANOSSY, G. (1972) Elicitation of Selective T and B Lymphocyte Responses by Cell Surface-binding Ligands. *Transplantn Rev.*, **11**, 87.

HELLSTRÖM, I. & HELLSTRÖM, K. (1970) Colony Inhibition and Cytotoxicity Assays. In In vitro *Methods in Cell-mediated Immunity.* Eds. B. R. Bloom and P. R. Glade. New York: Academic Press. p. 409.

HUNTER, W. M. & GREENWOOD, F. C. (1962) Preparation of Iodine-131 Labelled Human Growth Hormone of High Specific Activity. *Nature, Lond.*, **194**, 495.

JULIUS, M. H., SIMPSON, E. & HERZENBERG, L. A. (1973) A Rapid Method for the Isolation of Functional Thymus Derived Murine Lymphocytes. *Eur. J. Immun.*, **3**, 645.

KELLY, B., KAYE, B., YOSHIZAWA, W., LEVY, J. G. & KILBURN, D. G. (1974) Selective Binding of Chemically Defined Antigenic Peptides to Mouse Lymphocytes. *Eur. J. Immun.* In the press.

LAMON, E. W., SKURZAK, H. M., KLEIN, E. & WIGZELL, H. (1972) *In vitro* Cytotoxicity by a Nonthymus-processed Lymphocyte Population with Specificity for a Virally Determined Tumor Cell Surface Antigen. *J. exp. Med.*, **136**, 1072.

LAMON, E. W., WIGZELL, H., KLEIN, E., ANDERSON, B. & SKURZAK, H. M. (1973) The Lymphocyte Response to Primary Moloney Sarcoma Virus Tumours in Balb/c Mice. *J. exp. Med.*, **137**, 1472.

LECLERC, J. C., GOMARD, E., PLATA, F. & LEVY, J. P. (1973) Cell-mediated Immune Reaction Against Tumors Induced by Oncornaviruses. II. Nature of the Effector Cells in Tumor–Cell Cytolysis. *Int. J. Cancer*, **11**, 426.

OWEN, J. J. T. & SEEGER, R. C. (1973) Immunity to Tumours of the Murine Leukaemia–Sarcoma Virus Complex. *Br. J. Cancer*, **28**, Suppl. I, 26.

ROUSE, B. T., RÖLLINGHOFF, M. & WARNER, N. L. (1973) Tumor Immunity to Murine Plasma Cell Tumors. II. Essential Role of T Lymphocyte in Immune Response. *Eur. J. Immun.*, **3**, 218.

WHITNEY, R. B., LEVY, J. G. & SMITH, A. G. (1974) Influence of Tumor Size and Surgical Resection on Cell-mediated Immunity in Mice. *J. natn. Cancer Inst.*, **53**, 111.

WINN, H. J. (1959) The Immune Response and the Homograft Reaction. *Natn. Cancer Inst. Monog.*, **2**, 113.

ZARLING, J. M. & TEVETHIA, S. S. (1973) Transplantation Immunity to Simian Virus 40-transformed Cells in Tumour-bearing Mice II. Evidence for Macrophage Participation at the Effector Level of Tumour Cell Rejection. *J. natn. Cancer Inst.*, **50**, 149.